# Supplementary material for: 18F-FDG-PET/MR imaging to monitor disease activity in large vessel vasculitis
Source: Nat Commun. 2024 Aug 25;15:7314. doi: 10.1038/s41467-024-51613-1 (PMC11345444; doi:10.1038/s41467-024-51613-1)
Supplement: Supplementary file 1 — Supplementary Information [file 41467_2024_51613_MOESM1_ESM.pdf]

^{18}F-FDG-PET/MR imaging to monitor disease activity in large vessel vasculitis

Supplementary Material

CASE VIGNETTES

Case vignette 1

Patient Information

A 66-year-old female presented 3 years ago with a unilateral ‘jagging’ headache, left arm discomfort and constitutional symptoms including weight loss, reduced appetite, and night sweats. CRP and ESR were both elevated. PET/CT demonstrated aortic wall thickening and increased confluent FDG uptake in the thoracic/abdominal aorta, subclavian and carotid arteries which was greater than liver uptake, consistent with active large vessel vasculitis. She was diagnosed with LV-GCA and treated with glucocorticoid monotherapy.

Current Situation and Treatment

The patient now presents with worsening lethargy and fatigue. She describes an intermittent, bilateral headache which feels like a pressure. Her appetite and weight are maintained, and she has no other symptoms. Glucocorticoids were stopped a year ago.

Physical Examination

Blood pressure 114/84 mmHg (no significant difference between limbs).
Normal vascular examination with normal pulses bilaterally and no vascular bruits.
Normal temporal arteries on palpation.
Normal carotid arteries on palpation.
No other significant examination findings.

Laboratory Investigations

Haemoglobin:	140 g/L	(normal range 115-165 g/L)
White cell count:	8.6 $\times10^9$/L	(normal range 4.0-11.0 $\times10^9$/L)
Platelet count:	305 $\times10^9$/L	(normal range 150-400 $\times10^9$/L)
CRP:	21 mg/L	(normal range <10 mg/L)
ESR:	32 mm/hour	(normal range <30 mm/hour)

Questions:

1. Do you think this patient has active vasculitis?
    a. Yes
    b. No
    c. Not sure

2. How confident are you about this decision?
    a. Scale from 0 – 100

3. Would you like to alter immunosuppression at this point (currently no treatment)?
    a. Yes
    b. No
    c. Not sure

4. How confident are you about the decision to alter/not alter immunosuppression?
    a. Scale from 0 – 100

Case vignette 1 – Part 2

The patient undergoes a PET/MR scan which demonstrates:

- Unchanged aortic wall thickening (max 4mm)
- No areas of arterial stenosis, occlusion, or dilatation
- Normal mural T2 signal
- No post-GAD mural enhancement
- Low-grade, patchy FDG uptake in the thoracic/abdominal aorta, subclavian and carotid arteries which is less than liver uptake

Questions:

5. Having considered the PET/MR scan, does this change your opinion as to whether the patient has active vasculitis?
    a. Previously thought no, still think no
    b. Previously thought no, now think yes
    c. Previously thought no, now not sure
    d. Previously thought yes, still think yes
    e. Previously thought yes, now think no
    f. Previously thought yes, now not sure
    g. Previously not sure, still not sure
    h. Previously not sure, now think no
    i. Previously not sure, now think yes

6. How confident are you about this opinion?
    a. Scale from 0 – 100

7. Would you like to alter immunosuppression at this point?
    a. Previously thought no, still think no
    b. Previously thought no, now think yes
    c. Previously thought no, now not sure
    d. Previously thought yes, still think yes
    e. Previously thought yes, now think no
    f. Previously thought yes, now not sure
    g. Previously not sure, still not sure
    h. Previously not sure, now think no
    i. Previously not sure, now think yes

8. How confident are you about the decision to alter immunosuppression /keep immunosuppression the same?
    a. Scale from 0 - 100

Thank you, end of vignette.

Case vignette 2

Patient Information

A 38-year-old female presented 12 months ago with abdominal discomfort, altered bowel habit, reduced appetite and fatigue. CRP and ESR were both elevated. PET/CT demonstrated an area of aortic wall thickening and increased FDG uptake in the aortic arch which was greater than liver uptake. In addition, there was high-grade FDG uptake in the superior mesenteric artery (SMA) which was greater than liver uptake. CT angiography demonstrated a high-grade stenosis of the SMA. She was diagnosed with Takayasu arteritis and treated with prednisolone and tocilizumab.

Current Situation and Treatment

The patient now feels reasonably well but is still troubled with fatigue. Her abdominal pain has resolved, and her bowel habit and appetite have both normalised. She describes some niggling discomfort in her neck and left arm, but this is not preventing her from doing anything. She continues to take prednisolone and tocilizumab.

Physical Examination

Blood pressure 126/73 mmHg (no significant difference between limbs).
Normal vascular examination with normal pulses bilaterally and no vascular bruits.
Normal temporal arteries on palpation.
Normal carotid arteries on palpation.
No other significant examination findings.

Laboratory Investigations

Haemoglobin:	135 g/L	(normal range 115-165 g/L)
White cell count:	9.4 $x10^9$/L	(normal range 4.0-11.0 $x10^9$/L)
Platelet count:	240 $x10^9$/L	(normal range 150-400 $x10^9$/L)
CRP:	2 mg/L	(normal range <10 mg/L)
ESR:	12 mm/hour	(normal range <30 mm/hour)

Questions:

9. Do you think this patient has active vasculitis?
   a. Yes
   b. No
   c. Not sure

10. How confident are you about this decision?
    a. Scale from 0 – 100

11. Would you like to alter immunosuppression at this point (currently prednisolone 10mg daily and tocilizumab 162mg weekly)?
    a. Yes
    b. No
    c. Not sure

12. How confident are you about the decision to alter/not alter immunosuppression?
    a. Scale from 0 – 100

Case vignette 2 – Part 2

The patient undergoes a PET/MR scan which demonstrates:

- New area of abdominal aortic wall thickening (~5mm)
- Slight improvement in degree of SMA stenosis
- Increased mural T2 signal in aortic arch and abdominal aorta
- Post-GAD mural enhancement present in aortic arch and abdominal aorta
- Persistent moderate-grade FDG uptake in the aortic arch and a new area of high-grade FDG uptake in the abdominal aorta which is greater than liver FDG uptake and associated with new area of wall thickening
- Resolution of previously seen high-grade FDG uptake in the SMA

Questions:

13. Having considered the PET/MR scan, does this change your opinion as to whether the patient has active vasculitis?
    a. Previously thought no, still think no
    b. Previously thought no, now think yes
    c. Previously thought no, now not sure
    d. Previously thought yes, still think yes
    e. Previously thought yes, now think no
    f. Previously thought yes, now not sure
    g. Previously not sure, still not sure
    h. Previously not sure, now think no
    i. Previously not sure, now think yes

14. How confident are you about this opinion?
    a. Scale from 0 – 100

15. Would you like to alter immunosuppression at this point?
    a. Previously thought no, still think no
    b. Previously thought no, now think yes
    c. Previously thought no, now not sure
    d. Previously thought yes, still think yes
    e. Previously thought yes, now think no
    f. Previously thought yes, now not sure
    g. Previously not sure, still not sure
    h. Previously not sure, now think no
    i. Previously not sure, now think yes

16. How confident are you about the decision to alter immunosuppression /keep immunosuppression the same?
    a. Scale from 0 - 100

Thank you, end of vignette.

Case vignette 3

Patient Information

A 57-year-old male presented 2 years ago with fever, weight loss, night sweats, headache, and visual disturbance. CRP and ESR were both elevated with anaemia and thrombocytosis. Ultrasound of the temporal arteries was consistent with GCA, and MR angiography demonstrated confluent thickening of the aorta, carotid arteries and subclavian arteries with increased T2 signal and post-GAD enhancement. The patient was diagnosed with GCA and treated with prednisolone and methotrexate for a period of 12 months, and methotrexate alone thereafter.

Current Situation and Treatment

The patient now presents with loss of appetite, fatigue, bilateral shoulder pain and night sweats. Having regained the weight he initially lost, this has started to fall again. He has a mild headache but no visual disturbance.

Physical Examination

Blood pressure 152/95 mmHg (no significant difference between limbs).
Normal vascular examination with normal pulses bilaterally and no vascular bruits.
Thickened temporal arteries on palpation with normal pulsation.
Normal carotid arteries on palpation.
No other significant examination findings.

Laboratory Investigations

Haemoglobin:	105 g/L	(normal range 115-165 g/L)
White cell count:	14.6 $x10^9$/L	(normal range 4.0-11.0 $x10^9$/L)
Platelet count:	550 $x10^9$/L	(normal range 150-400 $x10^9$/L)
CRP:	65 mg/L	(normal range <10 mg/L)
ESR:	42 mm/hour	(normal range <30 mm/hour)

Questions:

17. Do you think this patient has active vasculitis?
    a. Yes
    b. No
    c. Not sure

18. How confident are you about this decision?
    a. Scale from 0 – 100

19. Would you like to alter immunosuppression at this point (currently methotrexate 15mg weekly)?
    a. Yes
    b. No
    c. Not sure

20. How confident are you about the decision to alter/not alter immunosuppression?
    a. Scale from 0 – 100

Case vignette 3 – Part 2

The patient undergoes a PET/MR scan which demonstrates:

- New, confluent aortic wall thickening (max 6mm)
- No areas of arterial stenosis, occlusion, or dilatation
- Increased mural T2 signal in the thoracic and abdominal aorta
- Post-GAD mural enhancement in the thoracic and abdominal aorta
- High-grade, confluent FDG uptake in the thoracic/abdominal aorta and carotid, subclavian, axillary, and vertebral arteries bilaterally which is greater than liver uptake

Questions:

21. Having considered the PET/MR scan, does this change your opinion as to whether the patient has active vasculitis?
    a. Previously thought no, still think no
    b. Previously thought no, now think yes
    c. Previously thought no, now not sure
    d. Previously thought yes, still think yes
    e. Previously thought yes, now think no
    f. Previously thought yes, now not sure
    g. Previously not sure, still not sure
    h. Previously not sure, now think no
    i. Previously not sure, now think yes

22. How confident are you about this opinion?
    a. Scale from 0 – 100

23. Would you like to alter immunosuppression at this point?
    a. Previously thought no, still think no
    b. Previously thought no, now think yes
    c. Previously thought no, now not sure
    d. Previously thought yes, still think yes
    e. Previously thought yes, now think no
    f. Previously thought yes, now not sure
    g. Previously not sure, still not sure
    h. Previously not sure, now think no
    i. Previously not sure, now think yes

24. How confident are you about the decision to alter immunosuppression /keep immunosuppression the same?
    a. Scale from 0 - 100

Thank you, end of vignette.

Case vignette 4

Patient Information

A 45-year-old female presented 4 years ago with fever, weight loss, chest pain and left arm claudication. The left radial pulse was palpable but weak, and there was a blood pressure discrepancy between arms. CRP and ESR were both marginally elevated. MR angiography demonstrated confluent thickening of the aorta, carotid arteries, and subclavian arteries bilaterally with increased T2 signal and post-GAD enhancement. There was a high-grade stenosis at the origin of the left subclavian artery. She was diagnosed with Takayasu arteritis and treated with prednisolone and mycophenolate mofetil for a period of 18 months, and mycophenolate mofetil alone thereafter.

Current Situation and Treatment

The patient now feels well. Her symptoms at time of presentation have all resolved other than mild residual left arm discomfort after intensive use. She has a mild tremor which has been present since starting treatment.

Physical Examination

Blood pressure 110/64 mmHg (no significant difference between limbs).
Normal vascular examination with normal pulses bilaterally and no vascular bruits.
Normal carotid arteries on palpation.
No other significant examination findings.

Laboratory Investigations

Haemoglobin:	138 g/L	(normal range 115-165 g/L)
White cell count:	7.2 $x10^9$/L	(normal range 4.0-11.0 $x10^9$/L)
Platelet count:	310 $x10^9$/L	(normal range 150-400 $x10^9$/L)
CRP:	1 mg/L	(normal range <10 mg/L)
ESR:	6 mm/hour	(normal range <30 mm/hour)

Questions:

25. Do you think this patient has active vasculitis?
    a. Yes
    b. No
    c. Not sure

26. How confident are you about this decision?
    a. Scale from 0 – 100

27. Would you like to alter immunosuppression at this point (currently mycophenolate mofetil 1g twice daily)?
    a. Yes
    b. No
    c. Not sure

28. How confident are you about the decision to alter/not alter immunosuppression?
    a. Scale from 0 – 100

Case vignette 4 – Part 2

The patient undergoes a PET/MR scan which demonstrates:

- Reduction in the degree of aortic wall thickening (max 3.5mm)
- Mild residual left subclavian artery stenosis
- Normal mural T2 signal throughout
- No post-GAD mural enhancement
- No significant arterial FDG uptake

Questions:

29. Having considered the PET/MR scan, does this change your opinion as to whether the patient has active vasculitis?
    a. Previously thought no, still think no
    b. Previously thought no, now think yes
    c. Previously thought no, now not sure
    d. Previously thought yes, still think yes
    e. Previously thought yes, now think no
    f. Previously thought yes, now not sure
    g. Previously not sure, still not sure
    h. Previously not sure, now think no
    i. Previously not sure, now think yes

30. How confident are you about this opinion?
    a. Scale from 0 – 100

31. Would you like to alter immunosuppression at this point?
    a. Previously thought no, still think no
    b. Previously thought no, now think yes
    c. Previously thought no, now not sure
    d. Previously thought yes, still think yes
    e. Previously thought yes, now think no
    f. Previously thought yes, now not sure
    g. Previously not sure, still not sure
    h. Previously not sure, now think no
    i. Previously not sure, now think yes

32. How confident are you about the decision to alter immunosuppression /keep immunosuppression the same?
    a. Scale from 0 - 100

Thank you, end of vignette.

Case vignette 5

Patient Information

A 24-year-old male presented 2 years ago with left arm claudication and chest discomfort. CRP and ESR were both elevated. MR angiogram at that time demonstrated circumferential wall thickening of the ascending aorta, aortic arch, left common carotid and left subclavian artery, with increased T2 mural signal and post-GAD mural enhancement in these areas. In addition, there was a proximal left subclavian artery stenosis with evidence of collateralisation. The patient was diagnosed with Takayasu arteritis and treated with prednisolone and infliximab for 18 months, and prednisolone monotherapy thereafter.

Current Situation and Treatment

The patient now reports a feeling of light-headedness and mild chest discomfort. He feels otherwise reasonably well. His left arm remains painful with repetitive use but has not changed.

Physical Examination

Blood pressure 132/76 mmHg right arm, 105/66 mmHg left arm.
Left radial pulse thready.
Bruit audible upper left chest wall.
Normal temporal arteries on palpation.
Normal carotid arteries on palpation.
No other significant examination findings.

Laboratory Investigations

Haemoglobin:	145 g/L	(normal range 115-165 g/L)
White cell count:	11.1 $x10^9$/L	(normal range 4.0-11.0 $x10^9$/L)
Platelet count:	280 $x10^9$/L	(normal range 150-400 $x10^9$/L)
CRP:	13 mg/L	(normal range <10 mg/L)
ESR:	16 mm/hour	(normal range <30 mm/hour)

Questions:

33. Do you think this patient has active vasculitis?
    a. Yes
    b. No
    c. Not sure

34. How confident are you about this decision?
    a. Scale from 0 – 100

35. Would you like to alter immunosuppression at this point (currently prednisolone 5mg)?
    a. Yes
    b. No
    c. Not sure

36. How confident are you about the decision to alter/not alter immunosuppression?
    a. Scale from 0 – 100

Case vignette 5 – Part 2

The patient undergoes a PET/MR scan which demonstrates:

- Worsened circumferential thickening of the ascending aorta, aortic arch, left common carotid and left subclavian artery with increased T2 mural signal and post-GAD mural enhancement in these areas
- No change in degree of left subclavian artery stenosis
- New left common carotid and left vertebral artery stenoses
- Moderate-grade confluent FDG uptake in the ascending aorta, aortic arch and carotids, subclavian and vertebral arteries bilaterally

Questions:

37. Having considered the PET/MR scan, does this change your opinion as to whether the patient has active vasculitis?
    a. Previously thought no, still think no
    b. Previously thought no, now think yes
    c. Previously thought no, now not sure
    d. Previously thought yes, still think yes
    e. Previously thought yes, now think no
    f. Previously thought yes, now not sure
    g. Previously not sure, still not sure
    h. Previously not sure, now think no
    i. Previously not sure, now think yes

38. How confident are you about this opinion?
    a. Scale from 0 – 100

39. Would you like to alter immunosuppression at this point?
    a. Previously thought no, still think no
    b. Previously thought no, now think yes
    c. Previously thought no, now not sure
    d. Previously thought yes, still think yes
    e. Previously thought yes, now think no
    f. Previously thought yes, now not sure
    g. Previously not sure, still not sure
    h. Previously not sure, now think no
    i. Previously not sure, now think yes

40. How confident are you about the decision to alter immunosuppression /keep immunosuppression the same?
    a. Scale from 0 - 100

Thank you, end of vignette.

Case vignette 6

Patient Information

A 41-year-old female presented 6 years ago with fatigue, chest discomfort, and shortness of breath. CRP and ESR were both elevated. PET/CT at that time demonstrated circumferential wall thickening of the aorta and increased FDG uptake throughout the aorta which was greater than liver uptake. In addition, there was dilatation of the aortic root and a stenotic section of descending thoracic aorta. Echocardiogram confirmed moderate left ventricular systolic dysfunction and moderate aortic regurgitation. The patient was diagnosed with Takayasu arteritis and treated with prednisolone and methotrexate for 2 years.

Current Situation and Treatment

The patient now presents with worsening shortness of breath and lethargy. She has developed ankle swelling and pain in her calves bilaterally.

Physical Examination

Blood pressure 135/45 mmHg (no significant difference between limbs).
Early diastolic murmur, loudest left lower sternal edge.
Otherwise normal vascular examination with normal pulses bilaterally and no vascular bruits.
Normal temporal arteries on palpation.
Normal carotid arteries on palpation.
Pitting oedema to mid-shin bilaterally.

Laboratory Investigations

Haemoglobin:	125 g/L	(normal range 115-165 g/L)
White cell count:	4.6 $\times10^9$/L	(normal range 4.0-11.0 $\times10^9$/L)
Platelet count:	320 $\times10^9$/L	(normal range 150-400 $\times10^9$/L)
CRP:	22 mg/L	(normal range <10 mg/L)
ESR:	35 mm/hour	(normal range <30 mm/hour)

Questions:

41. Do you think this patient has active vasculitis?
    a. Yes
    b. No
    c. Not sure

42. How confident are you about this decision?
    a. Scale from 0 – 100

43. Would you like to alter immunosuppression at this point (currently no treatment)?
    a. Yes
    b. No
    c. Not sure

44. How confident are you about the decision to alter/not alter immunosuppression?
    a. Scale from 0 – 100

Case vignette 6 – Part 2

The patient undergoes a PET/MR scan which demonstrates:

- Reduction in the degree of aortic wall thickening
- Normal mural T2 signal throughout
- No post-GAD mural enhancement
- Further dilatation of aortic root
- No change in degree of descending thoracic aorta stenosis
- No significant arterial FDG uptake

Questions:

45. Having considered the PET/MR scan, does this change your opinion as to whether the patient has active vasculitis?
    a. Previously thought no, still think no
    b. Previously thought no, now think yes
    c. Previously thought no, now not sure
    d. Previously thought yes, still think yes
    e. Previously thought yes, now think no
    f. Previously thought yes, now not sure
    g. Previously not sure, still not sure
    h. Previously not sure, now think no
    i. Previously not sure, now think yes

46. How confident are you about this opinion?
    a. Scale from 0 – 100

47. Would you like to alter immunosuppression at this point?
    a. Previously thought no, still think no
    b. Previously thought no, now think yes
    c. Previously thought no, now not sure
    d. Previously thought yes, still think yes
    e. Previously thought yes, now think no
    f. Previously thought yes, now not sure
    g. Previously not sure, still not sure
    h. Previously not sure, now think no
    i. Previously not sure, now think yes

48. How confident are you about the decision to alter immunosuppression /keep immunosuppression the same?
    a. Scale from 0 - 100

Thank you, end of vignette.

Case vignette 7

Patient Information

A 78-year-old female presented 12 months ago with left arm discomfort and constitutional symptoms including fever, weight loss and profound fatigue. CRP and ESR were both elevated. PET/CT demonstrated aortic wall thickening and increased confluent FDG uptake in the thoracic/abdominal aorta, subclavian and axillary arteries which was greater than liver uptake, consistent with active large vessel vasculitis. In addition, CT angiogram, demonstrated a 3cm left axillary artery stenosis with evidence of collateralisation. She was diagnosed with LV-GCA and treated with prednisolone and mycophenolate mofetil and remains on both.

Current Situation and Treatment

The patient currently feels reasonably well, although is still troubled by fatigue. She also describes a feeling of weakness in her thighs bilaterally and a mild tremor. She has regained the weight she lost. Her left arm discomfort is unchanged.

Physical Examination

Blood pressure 180/105 mmHg right, 160/100 mmHg on left.
Left radial pulse absent, otherwise normal pulses.
Normal temporal arteries on palpation.
Normal carotid arteries on palpation.
No other significant examination findings.

Laboratory Investigations

Haemoglobin:	118 g/L	(normal range 115-165 g/L)
White cell count:	12.9 $x10^9$/L	(normal range 4.0-11.0 $x10^9$/L)
Platelet count:	380 $x10^9$/L	(normal range 150-400 $x10^9$/L)
CRP:	2 mg/L	(normal range <10 mg/L)
ESR:	11 mm/hour	(normal range <30 mm/hour)

Questions:

49. Do you think this patient has active vasculitis?
    a. Yes
    b. No
    c. Not sure

50. How confident are you about this decision?
    a. Scale from 0 – 100

51. Would you like to alter immunosuppression at this point (currently prednisolone 5mg daily and mycophenolate mofetil 500mg twice daily)?
    a. Yes
    b. No
    c. Not sure

52. How confident are you about the decision to alter/not alter immunosuppression?
    a. Scale from 0 – 100

Case vignette 7 – Part 2

The patient undergoes a PET/MR scan which demonstrates:

- Unchanged aortic wall thickening
- Normal mural T2 signal
- No post-GAD mural enhancement
- Low-grade, patchy FDG uptake in the thoracic/abdominal aorta which is less than liver uptake
- No change in left axillary artery stenosis

Questions:

53. Having considered the PET/MR scan, does this change your opinion as to whether the patient has active vasculitis?
    a. Previously thought no, still think no
    b. Previously thought no, now think yes
    c. Previously thought no, now not sure
    d. Previously thought yes, still think yes
    e. Previously thought yes, now think no
    f. Previously thought yes, now not sure
    g. Previously not sure, still not sure
    h. Previously not sure, now think no
    i. Previously not sure, now think yes

54. How confident are you about this opinion?
    a. Scale from 0 – 100

55. Would you like to alter immunosuppression at this point?
    a. Previously thought no, still think no
    b. Previously thought no, now think yes
    c. Previously thought no, now not sure
    d. Previously thought yes, still think yes
    e. Previously thought yes, now think no
    f. Previously thought yes, now not sure
    g. Previously not sure, still not sure
    h. Previously not sure, now think no
    i. Previously not sure, now think yes

56. How confident are you about the decision to alter immunosuppression /keep immunosuppression the same?
    a. Scale from 0 - 100

Thank you, end of vignette.

Case vignette 8

Patient Information

An 81-year-old male presented 18 months ago with headache, jaw discomfort, visual disturbance and constitutional symptoms including fever, fatigue, and reduced appetite. CRP and ESR were both elevated. Ultrasound demonstrated bilateral thickening of the superficial temporal arteries and axillary arteries with 'halo' sign. CT demonstrated thickening of the aortic wall, and subclavian and axillary arteries bilaterally. He was diagnosed with GCA and treated with prednisolone initially, and tocilizumab was added after six months. He remains on both.

Current Situation and Treatment

The patient currently feels better but remains fatigued. His appetite and headache have improved, and his fevers have gone. He describes an intermittent chest discomfort which radiates to his back.

Physical Examination

Blood pressure 174/95 mmHg (no significant difference between limbs).
Normal vascular examination with normal pulses bilaterally and no vascular bruits.
Normal temporal arteries on palpation.
Normal carotid arteries on palpation.
No other significant examination findings.

Laboratory Investigations

Haemoglobin:	115 g/L	(normal range 115-165 g/L)
White cell count:	14.1 x10^9/L	(normal range 4.0-11.0 x10^9/L)
Platelet count:	380 x10^9/L	(normal range 150-400 x10^9/L)
CRP:	0 mg/L	(normal range <10 mg/L)
ESR:	8 mm/hour	(normal range <30 mm/hour)

Questions:

57. Do you think this patient has active vasculitis?
    a. Yes
    b. No
    c. Not sure

58. How confident are you about this decision?
    a. Scale from 0 – 100

59. Would you like to alter immunosuppression at this point (currently prednisolone 5mg and tocilizumab 162mg weekly)?
    a. Yes
    b. No
    c. Not sure

60. How confident are you about the decision to alter/not alter immunosuppression?
    a. Scale from 0 – 100

Case vignette 8 – Part 2

The patient undergoes a PET/MR scan which demonstrates:

- Further thickening of the aortic wall
- No areas of arterial stenosis, occlusion, or dilatation
- Increased mural T2 signal in the thoracic and abdominal aorta
- Post-GAD mural enhancement in the thoracic and abdominal aorta
- Moderate-grade, confluent FDG uptake in the thoracic/abdominal aorta and subclavian and axillary arteries bilaterally which is greater than liver uptake

Questions:

61. Having considered the PET/MR scan, does this change your opinion as to whether the patient has active vasculitis?
    a. Previously thought no, still think no
    b. Previously thought no, now think yes
    c. Previously thought no, now not sure
    d. Previously thought yes, still think yes
    e. Previously thought yes, now think no
    f. Previously thought yes, now not sure
    g. Previously not sure, still not sure
    h. Previously not sure, now think no
    i. Previously not sure, now think yes

62. How confident are you about this opinion?
    a. Scale from 0 – 100

63. Would you like to alter immunosuppression at this point?
    a. Previously thought no, still think no
    b. Previously thought no, now think yes
    c. Previously thought no, now not sure
    d. Previously thought yes, still think yes
    e. Previously thought yes, now think no
    f. Previously thought yes, now not sure
    g. Previously not sure, still not sure
    h. Previously not sure, now think no
    i. Previously not sure, now think yes

64. How confident are you about the decision to alter immunosuppression /keep immunosuppression the same?
    a. Scale from 0 - 100

Thank you, end of vignette.

Case vignette 9

Patient Information

A 73-year-old male presented 6 months ago with headache, scalp tenderness, and constitutional symptoms including myalgia, and weight loss. CRP and ESR were both elevated. Temporal artery biopsy confirmed the diagnosis of GCA. CT demonstrated thickening of the aortic wall. He was treated with prednisolone monotherapy.

Current Situation and Treatment

The patient presents with an acute anterior circulation stroke. CT head confirms a right middle cerebral artery (MCA) infarct. He receives thrombolysis and makes a good initial recovery. He has a mild headache but no scalp tenderness.

Physical Examination

Blood pressure 155/92 mmHg (no significant difference between limbs).
Normal vascular examination with normal pulses bilaterally and no vascular bruits.
Normal temporal arteries on palpation.
Normal carotid arteries on palpation.
No other significant examination findings.

Laboratory Investigations

Haemoglobin:	124 g/L	(normal range 115-165 g/L)
White cell count:	13.6 $\times 10^9$/L	(normal range 4.0-11.0 $\times 10^9$/L)
Platelet count:	325 $\times 10^9$/L	(normal range 150-400 $\times 10^9$/L)
CRP:	32 mg/L	(normal range <10 mg/L)
ESR:	41 mm/hour	(normal range <30 mm/hour)

Questions:

65. Do you think this patient has active vasculitis?
    a. Yes
    b. No
    c. Not sure

66. How confident are you about this decision?
    a. Scale from 0 – 100

67. Would you like to alter immunosuppression at this point (currently prednisolone 15mg daily)?
    a. Yes
    b. No
    c. Not sure

68. How confident are you about the decision to alter/not alter immunosuppression?
    a. Scale from 0 – 100

Case vignette 9 – Part 2

The patient undergoes a PET/MR scan which demonstrates:

- Further thickening of the aortic wall and concentric thickening of the carotid and subclavian arteries bilaterally
- Increased mural T2 signal in the thoracic and abdominal aorta
- Post-GAD mural enhancement in the thoracic and abdominal aorta, and carotid arteries bilaterally
- Moderate-grade, confluent FDG uptake in the thoracic/abdominal aorta and carotid and subclavian arteries bilaterally which is greater than liver uptake

Questions:

69. Having considered the PETMR scan, does this change your opinion as to whether the patient has active vasculitis?
    a. Previously thought no, still think no
    b. Previously thought no, now think yes
    c. Previously thought no, now not sure
    d. Previously thought yes, still think yes
    e. Previously thought yes, now think no
    f. Previously thought yes, now not sure
    g. Previously not sure, still not sure
    h. Previously not sure, now think no
    i. Previously not sure, now think yes

70. How confident are you about this opinion?
    a. Scale from 0 – 100

71. Would you like to alter immunosuppression at this point?
    a. Previously thought no, still think no
    b. Previously thought no, now think yes
    c. Previously thought no, now not sure
    d. Previously thought yes, still think yes
    e. Previously thought yes, now think no
    f. Previously thought yes, now not sure
    g. Previously not sure, still not sure
    h. Previously not sure, now think no
    i. Previously not sure, now think yes

72. How confident are you about the decision to alter immunosuppression /keep immunosuppression the same?
    a. Scale from 0 - 100

Thank you, end of vignette.

Case vignette 10

Patient Information

A 36-year-old male presented 5 years ago with dizziness, fatigue, and weight loss. CRP and ESR were both elevated. CT angiogram at that time demonstrated circumferential wall thickening of the thoracic aorta and origins of the brachiocephalic artery, left common carotid, and left subclavian artery. In addition, there was a proximal left carotid artery stenosis. He was diagnosed with Takayasu arteritis and treated with prednisolone and methotrexate for 12 months, and then treatment was discontinued.

Current Situation and Treatment

The patient now presents with headaches and mild chest discomfort. He feels otherwise reasonably well. He has regained the weight he lost but still feels fatigued.

Physical Examination

Blood pressure 210/140 mmHg (no significant difference between limbs).
Normal vascular examination with normal pulses bilaterally and no vascular bruits.
Normal temporal arteries on palpation.
Normal carotid arteries on palpation.
Evidence of hypertensive retinopathy.

Laboratory Investigations

Haemoglobin:	160 g/L	(normal range 115-165 g/L)
White cell count:	5.1 $\times 10^9$/L	(normal range 4.0-11.0 $\times 10^9$/L)
Platelet count:	280 $\times 10^9$/L	(normal range 150-400 $\times 10^9$/L)
CRP:	14 mg/L	(normal range <10 mg/L)
ESR:	24 mm/hour	(normal range <30 mm/hour)

Questions:

73. Do you think this patient has active vasculitis?
    a. Yes
    b. No
    c. Not sure

74. How confident are you about this decision?
    a. Scale from 0 – 100

75. Would you like to alter immunosuppression at this point (currently no treatment)?
    a. Yes
    b. No
    c. Not sure

76. How confident are you about the decision to alter/not alter immunosuppression?
    a. Scale from 0 – 100

Case vignette 10 – Part 2

The patient undergoes a PET/MR scan which demonstrates:

- Improved circumferential wall thickening of the thoracic aorta and origins of the brachiocephalic artery, left common carotid, and left subclavian artery
- No change in degree of left proximal carotid artery stenosis.
- New tight left renal artery stenosis
- No significant arterial FDG uptake

Questions:

77. Having considered the PET/MR scan, does this change your opinion as to whether the patient has active vasculitis?
    a. Previously thought no, still think no
    b. Previously thought no, now think yes
    c. Previously thought no, now not sure
    d. Previously thought yes, still think yes
    e. Previously thought yes, now think no
    f. Previously thought yes, now not sure
    g. Previously not sure, still not sure
    h. Previously not sure, now think no
    i. Previously not sure, now think yes

78. How confident are you about this opinion?
    a. Scale from 0 – 100

79. Would you like to alter immunosuppression at this point?
    a. Previously thought no, still think no
    b. Previously thought no, now think yes
    c. Previously thought no, now not sure
    d. Previously thought yes, still think yes
    e. Previously thought yes, now think no
    f. Previously thought yes, now not sure
    g. Previously not sure, still not sure
    h. Previously not sure, now think no
    i. Previously not sure, now think yes

80. How confident are you about the decision to alter immunosuppression/keep immunosuppression the same?
    a. Scale from 0 - 100

Thank you, end of vignette.

SUPPLEMENTARY TABLES

Supplementary table 1. Assessment of disease activity

	**Baseline scan**		**Follow-up scan**		
	Active disease based on clinician assessment	Inactive disease based on clinician assessment	Active disease based on clinician assessment	Inactive disease based on clinician assessment	Total
Active disease based on qualitative radiologist assessment	14	1	4	1	20
Inactive disease based on qualitative radiologist assessment	3	6	2	9	20
Total	17	7	6	10	40

Supplementary table 2. Univariable analyses assessing ability of PET quantification methods to predict active *versus* inactive disease.

Estimates for all predictors are per 1 unit increase unless otherwise stated. SUV_{mean} TBR combined aortic score has been transformed (x10^1) for clarity. Two-sided analyses. *AICc, Akaike's corrected information criterion; CI, confidence interval; NPV, negative predictive value; OR, odds ratio; PETVAS, PET vasculitis activity score; PPV, positive predictive value; SUV, standardised uptake value; TBR, target-to-background ratio*

Predictor	Range	Estimate (95% CI)	AICc	PPV	NPV	*P* value
**Visual comparison with liver**						
Total score (PETVAS)	3 – 27	1.2 (1.1 – 1.5)	46.4	76.2	66.7	**0.003**
**SUV_{max} TBR**						
Combined aortic score	5.1 – 17.0	1.5 (1.1 – 2.2)	51.7	72.0	71.4	**0.01**
Combined great vessel score	10.5 – 28.3	1.4 (1.1 – 1.9)	50.3	68.2	58.8	**0.02**
Total score	15.7 – 40.8	1.3 (1.1 – 1.6)	48.7	73.9	68.8	**0.01**
**SUV_{mean} TBR**						
Combined aortic score (x10^1)	0 – 46	1.4 (1.1 – 1.7)	38.9	82.6	81.3	**0.0001**
Combined great vessel score	0 – 8.1	3.7 (1.6 – 12.4)	45.4	83.3	66.7	**0.002**
Total score	0.3 – 12.7	5.1 (2.1 – 18.7)	36.3	90	79.0	**<0.0001**

Supplementary table 3. Univariable analyses assessing ability of MRI metrics to predict active *versus* inactive disease.

Estimates for all predictors are per 1 unit increase unless otherwise stated. Two-sided analyses. *AICc, Akaike's corrected information criterion; CI, confidence interval; NPV, negative predictive value; OR, odds ratio; PPV, positive predictive value.*

Predictor	Range	Estimate (95% CI)	AICc	PPV	NPV	*P* value
**Mural signal**						
Any arterial segment with increased T2-weighted mural signal	-	11.1 (1.7 – 218.1)	50.7	90.0	55.2	0.06
Total arterial segments with increased T2-weighted mural signal	0 – 11	2.4 (1.3 – 9.7)	47.2	90.9	57.1	**0.03**
**Mural enhancement**						
Any arterial segment with mural enhancement	-	1.8 (0.5 – 7.0)	56.9	66.7	45.0	0.4
Total arterial segments with mural enhancement	0 – 9	1.5 (1.0 – 2.6)	53.9	64.7	50.0	0.2
**Mural thickness**						
Maximum aortic mural thickness (per 1mm increase)	2.4 – 8.2	0.9 (0.6 – 1.5)	57.7	56.8	50.0	0.9
Total arterial segments with increased mural thickness	0 – 10	1.3 (1.0 – 1.8)	54.5	61.3	62.5	0.2
**Luminal abnormalities**						
Total territories with luminal abnormality	0 - 7	1.1 (0.7 – 1.8)	57.7	56.4	-	0.4

Supplementary table 4. Multivariable analyses assessing ability of multiple PET/MR parameters to predict active *versus* inactive disease

Estimates for all predictors are per 1 unit increase unless otherwise stated. Two-sided analyses. *AICc, Akaike's corrected information criterion; AUC, area under the curve; CI, confidence interval; NPV, negative predictive value; OR, odds ratio; PPV, positive predictive value; SUV, standardised uptake value; TBR, target-to-background ratio.*

**Model**	**Estimate (95% CI)**	**AICc**	**PPV**	**NPV**	**AUC**	**VIF**	***P* value**
**Model 1**							
Total SUV_{mean} TBR score	5.1 (2.1 – 18.7)	36.3	90.0	79.0	0.91	-	<0.0001
**Model 2**							
Total SUV_{mean} TBR score	4.5 (1.8 – 17.3)						
+ Total arterial segments with increased T2-weighted mural signal	2.0 (0.9 – 7.6)	35.7	86.4	82.4	0.93	2.5	<0.0001
**Model 3**							
Total SUV_{mean} TBR score	6.4 (1.9 – 45.5)						
+ Total arterial segments with increased T2-weighted mural signal	2.1 (1.0 – 8.0)						
+ PETVAS	0.9 (0.6 – 1.2)	37.6	86.4	82.4	0.93	3.6	<0.0001
**Model 4**							
Total SUV_{mean} TBR score	9.0 (2.1 – 91.6)						
+ Total arterial segments with increased T2-weighted mural signal	2.3 (1.0 – 11.8)						
+ Total SUV_{max} TBR score	0.8 (0.5 – 1.1)	36.7	90.5	83.3	0.95	3.6	<0.0001

Supplementary table 5. Outline of PET/MRI protocol.

MRI sequence	Reason for inclusion	Parameters
Standard coronal 3D Dixon VIBE for MRAC	Attenuation correction only	TR/TE1/TE2/α = 3.6ms/1.23ms/2.46ms/10º<br>FOV = 280 x 230<br>Matrix = 192 x 79<br>Slice thickness = 3mm<br>PE steps = 117
Transverse black blood HASTE	Anatomical assessment	TR/TE/α = dependent on R-R interval (~900 ms)/32ms/147º)<br>FOV = 352 x 512<br>Matrix = 256 x 104<br>Slice thickness = 8mm<br>PE steps = 61
ECG-gated coronal T2 TSE	Assessment of T2-weighted mural signal	TR/TE/TI/α = dependent on R-R interval (>6000ms)/ 53ms/220ms/120 º<br>FOV= 400 x 260<br>Matrix = 256 x 158<br>Slice thickness = 6 mm<br>PE steps = 298
Single slice areas of interest using breath-held and ECG-gated black blood 2D FLASH	High-definition anatomical assessment, useful for specific areas of interest and calculation of mural thickness	TR/TE/α = dependent on R-R interval/ 2.91ms/ 30º<br>FOV = 208 x 256<br>Matrix = 256 x 187<br>Slice thickness = 5 mm<br>PE steps = 192
Pre- and post-contrast breath-held transverse T1 VIBE	3D anatomical assessment and assessment of post-contrast mural enhancement	TR/TE/α= 4.00ms/ 1.85ms/ 5º<br>FOV = 252 x 384<br>Matrix = 384 x 189<br>Slice thickness = 3mm<br>PE steps = 215
2-bed MR angiogram using coronal 3D FLASH	Assessment of luminal abnormalities including stenosis, occlusion, dilatation, and aneurysm	TR/TE/α = 2.94 s/ 1.03ms/25º<br>FOV = 384 x 312<br>Matrix = 384 x 256<br>Slice thickness = 1.1 mm<br>PE steps = 245

SUPPLEMENTARY FIGURES

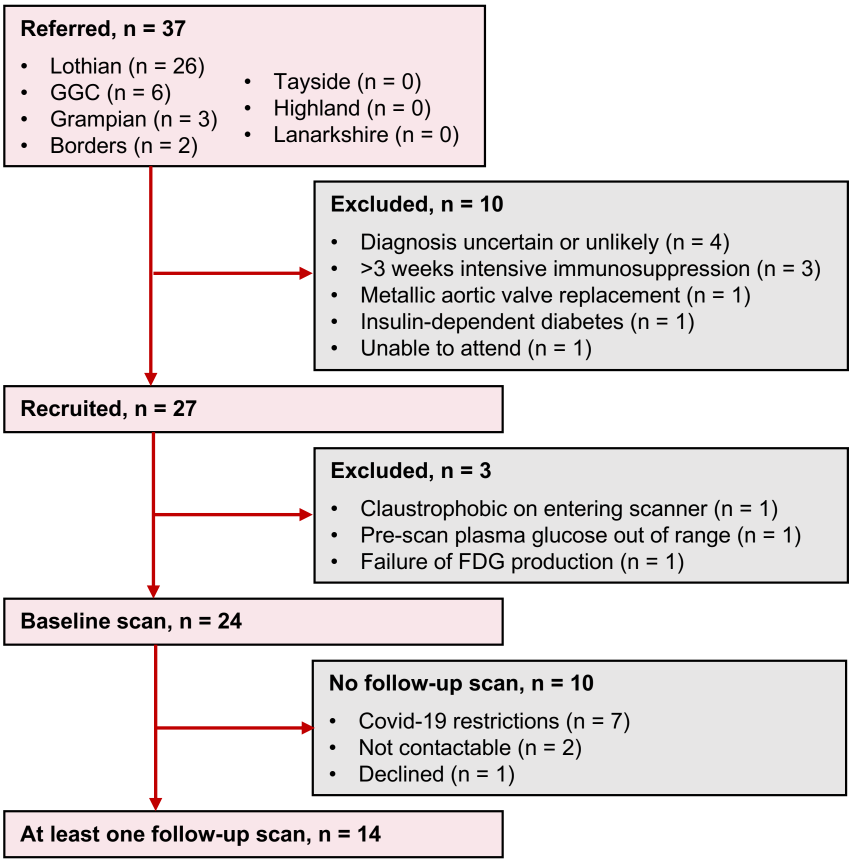

Supplementary figure 1. Consort diagram. *FDG, ^{18}F-fluorodeoxyglucose; GGC, Greater Glasgow and Clyde.*

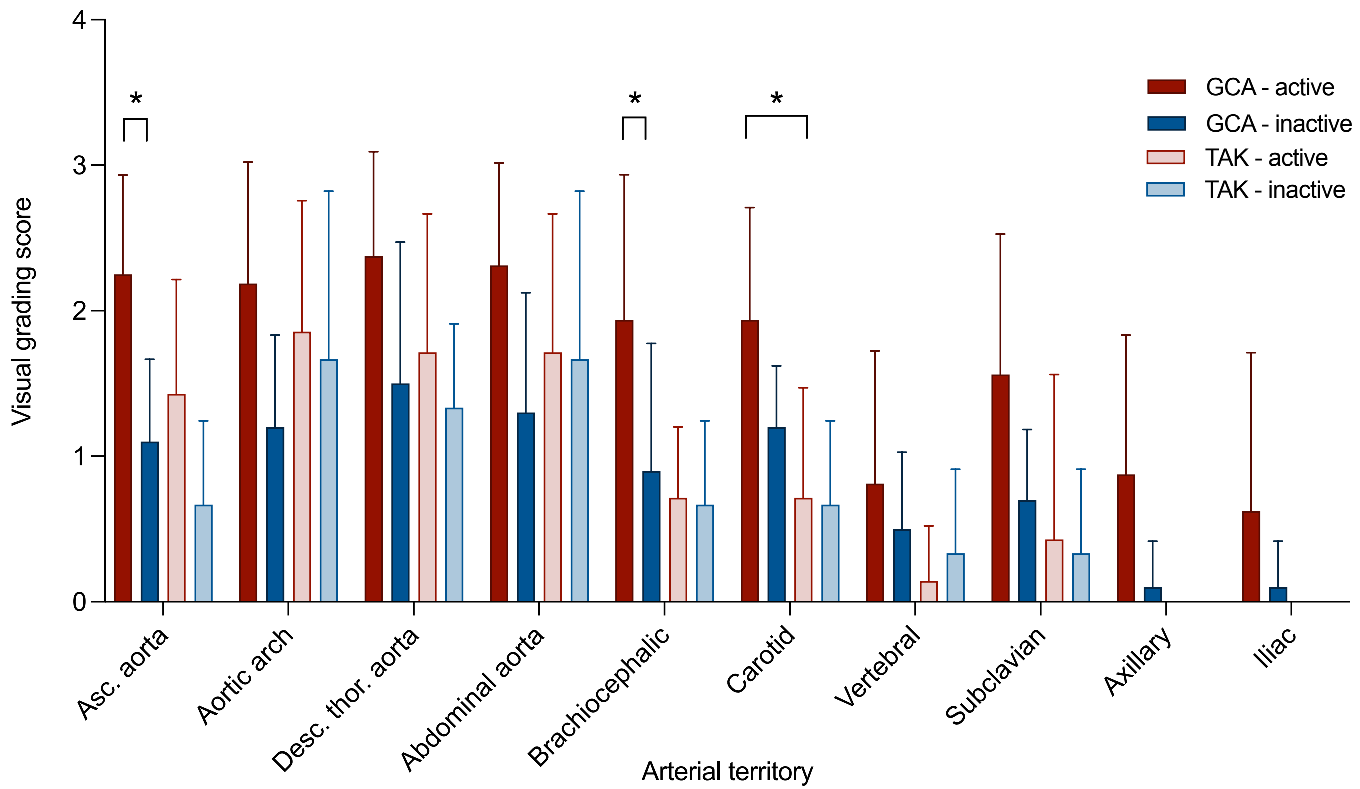

Supplementary figure 2. Visual assessment of disease activity in GCA *vs.* TAK. Degree of FDG uptake based on visual comparison with the liver in those with GCA (active/inactive disease, *n*=26) and TAK (active/inactive disease, *n*=10). FDG uptake was greater in active GCA *vs.* active TAK in the carotid arteries only (*P*=0.03). Data are presented as mean values +/- SD. Analysis by two-way ANOVA with Šidák's multiple comparison test. *Asc. aorta, ascending aorta; desc. thor. aorta, descending thoracic aorta.*

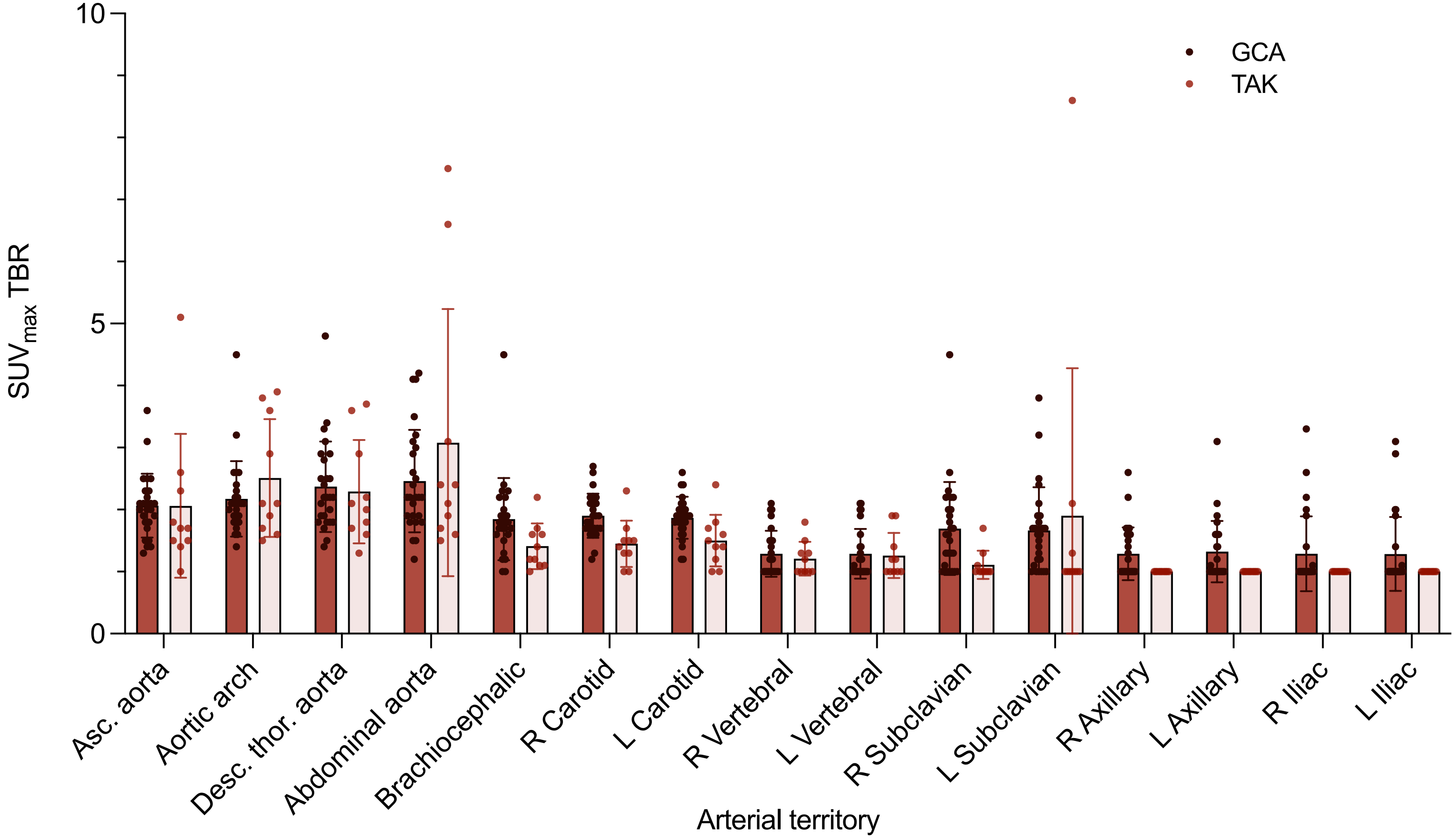

Supplementary figure 3. Degree of FDG uptake based on SUV$_{max}$ TBR analysis in those with GCA (*n*=26) *versus* TAK (*n*=10). No difference was observed in any arterial territory. Data are presented as mean values +/- SD. Analysis by two-way ANOVA with Šidák's multiple comparison test.

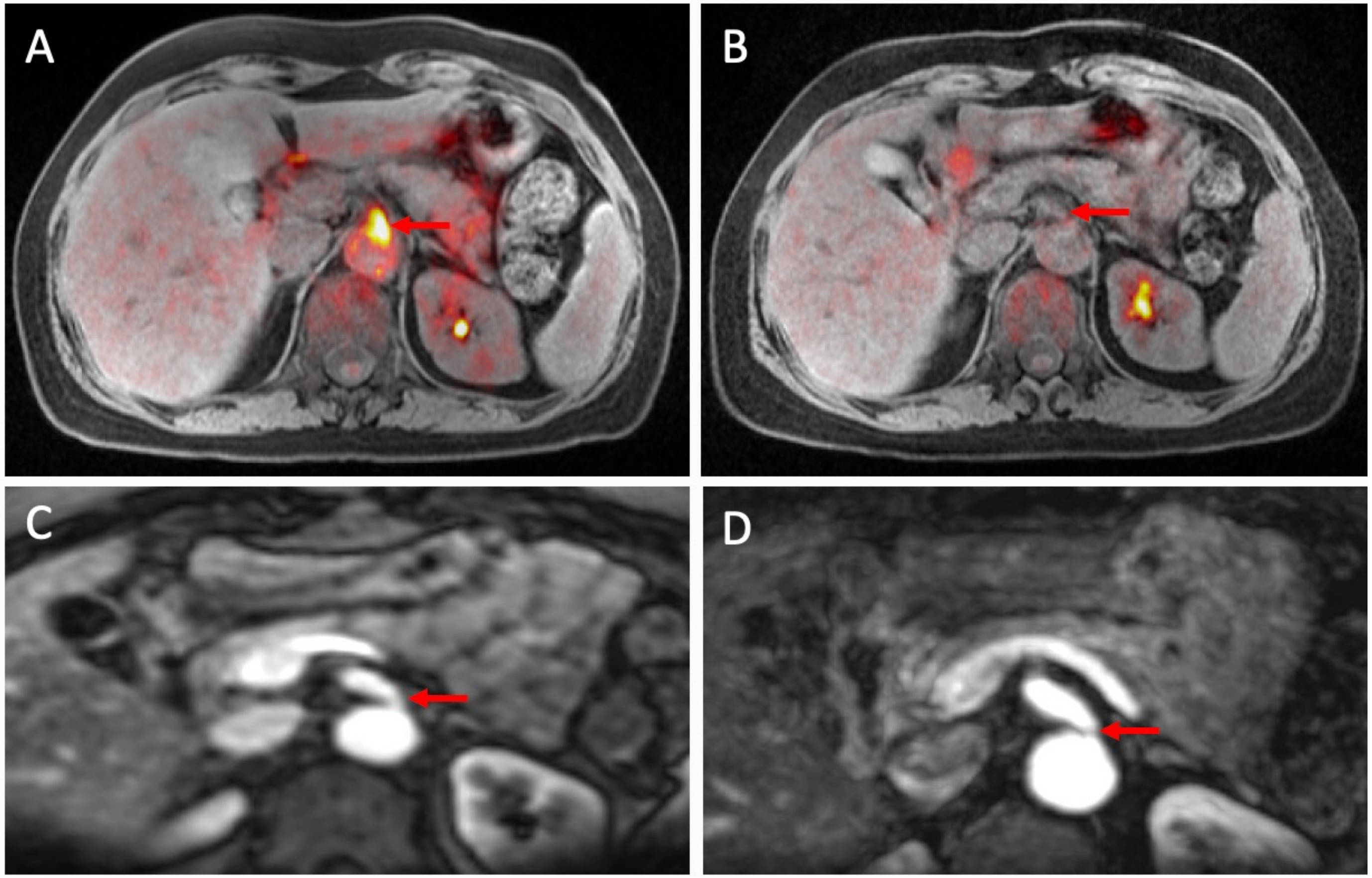

Supplementary figure 4. Example of reduction in FDG uptake between baseline and follow-up with no corresponding angiographic change. Panel A is a PET/MR image obtained at baseline demonstrating high grade FDG uptake affecting the origin of the superior mesenteric artery (arrow). Panel B shows resolution of FDG uptake at follow-up. Panel C demonstrates an area of stenosis at the origin of the superior mesenteric artery at baseline which is unchanged at time of follow-up (Panel D) despite apparent resolution of inflammation.

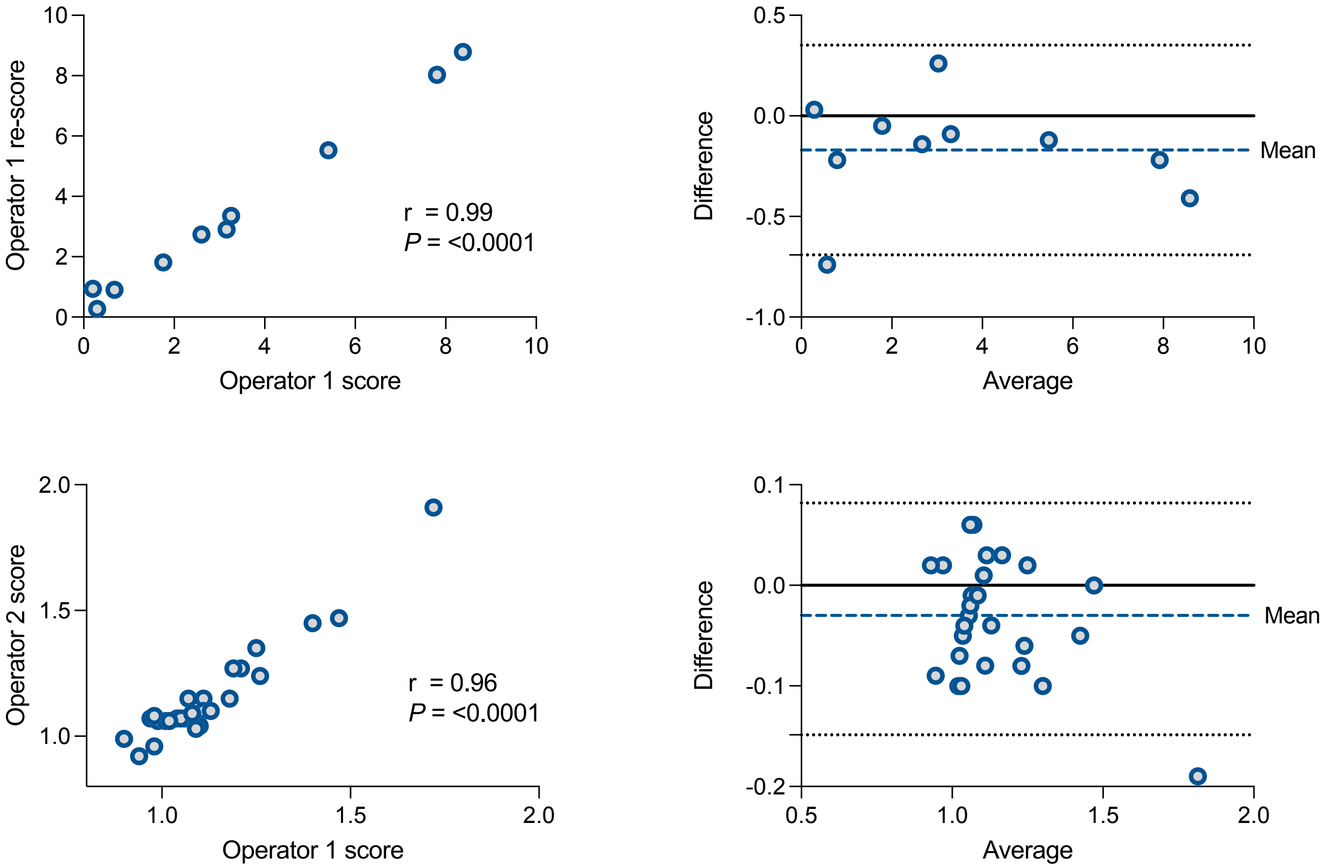

Supplementary figure 5. Intra- and inter-operator reliability of the VAMP score. A high degree of reliability was observed between independent operators (bottom graphs, *n*=26) and when the same operator re-scored the VAMP score on a randomly selected group of scans (top graphs, *n*=10). Correlations performed using two-sided Pearson's correlation coefficient. Bland-Altman plots demonstrate mean (or bias) (dashed blue line), and 95% limits of agreement (dotted black lines).

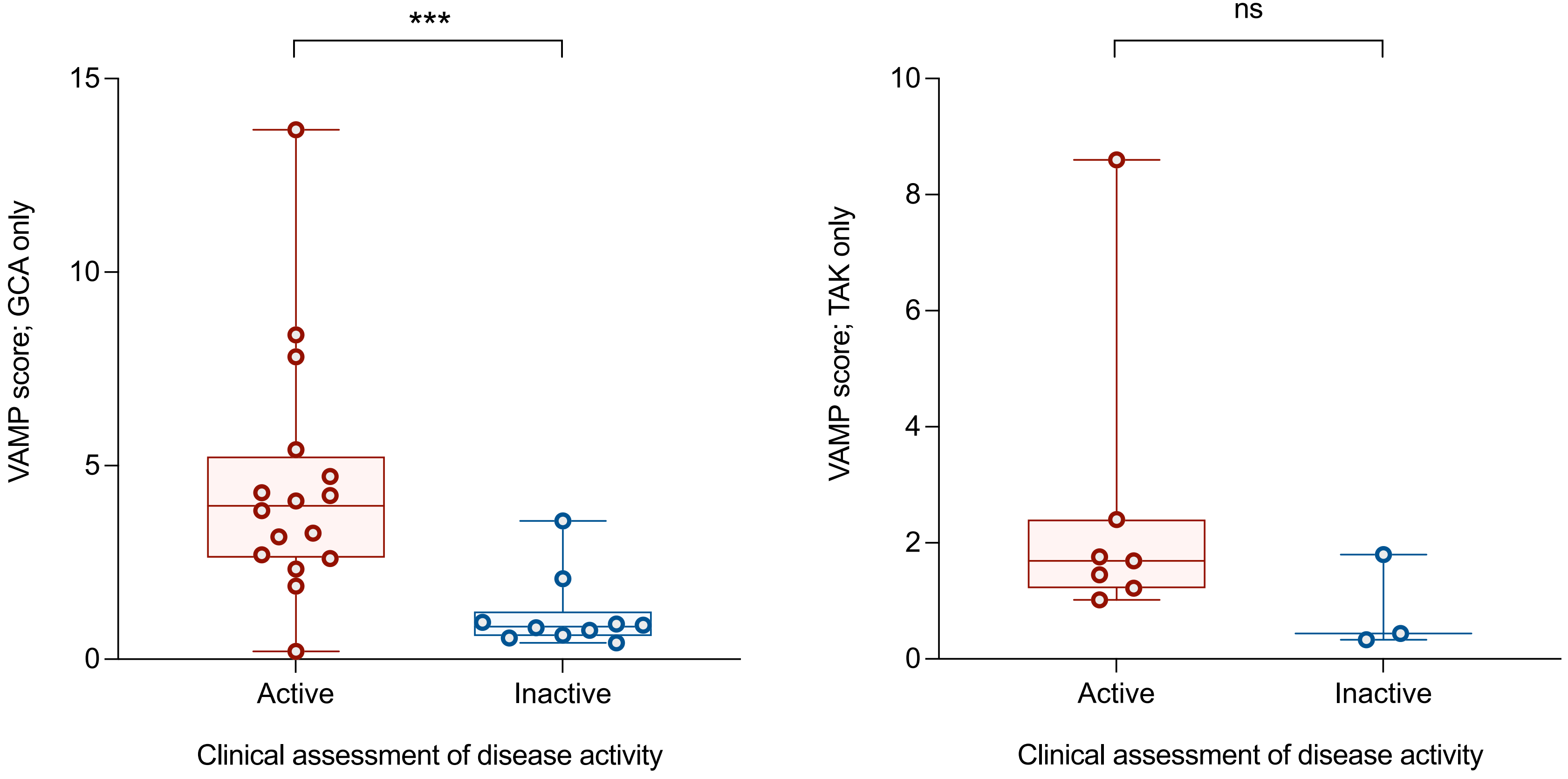

Supplementary figure 6. VAMP score values in participants with GCA (*n*=26, *P*=0.0004) and TAK (*n*=10) (active *versus* inactive disease). For box and whisker plots, the central line represents the median, the box represents the interquartile range, and the whiskers represent minimum and maximum values. Analyses by two-sided Mann-Whitney test.

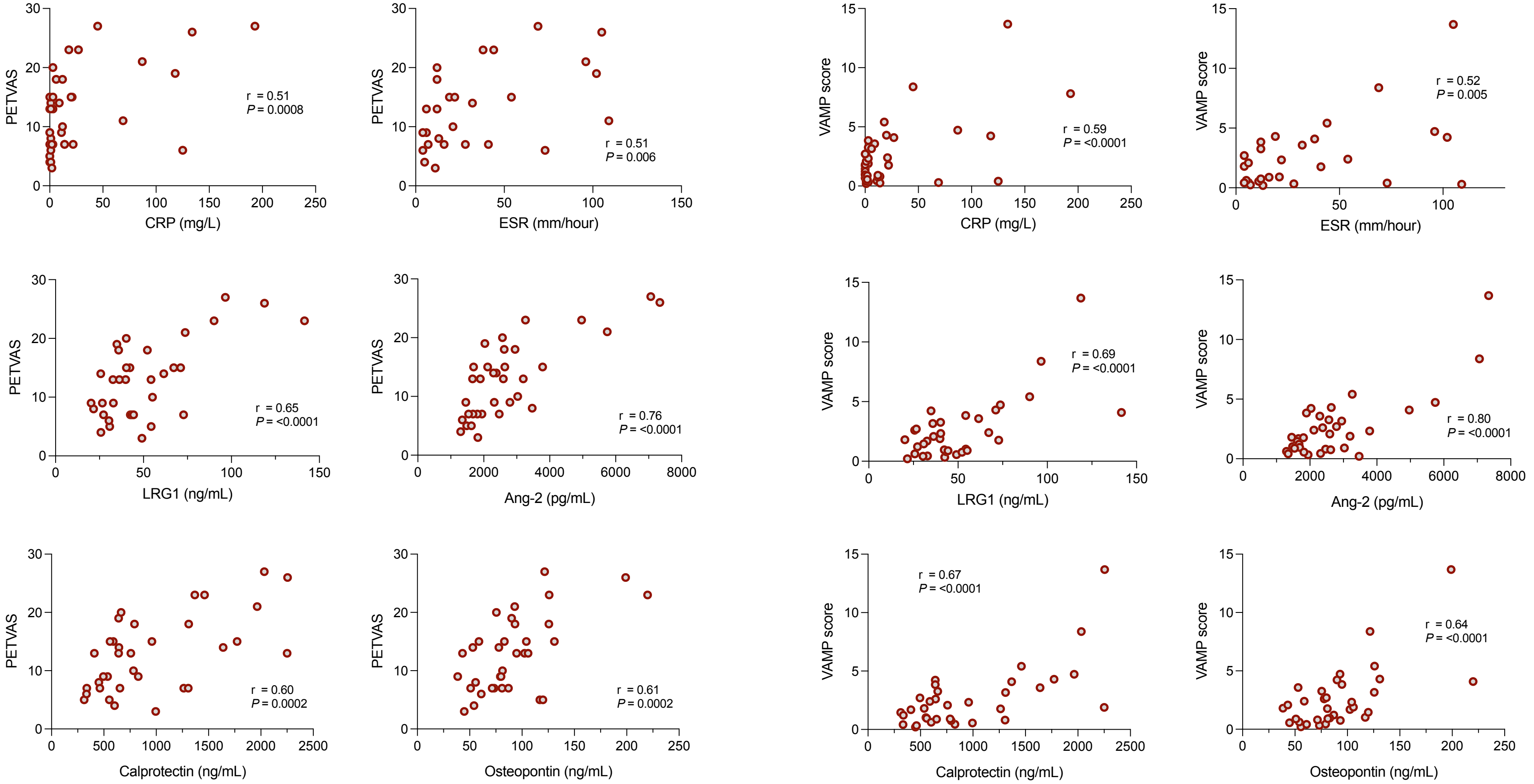

Supplementary figure 7. **Correlations between VAMP score and PETVAS, and serological markers of disease activity.** Serological markers include the emerging biomarkers LRG1, Ang-2, calprotectin, and osteopontin *(n*=39). Analysis by two-sided Pearson's correlation coefficient. Ang-2, angiopoietin-2; *CRP, C-reactive protein; ESR, erythrocyte sedimentation rate; LRG1, leucine-rich α-2 glycoprotein 1; PETVAS, PET vasculitis activity score.*

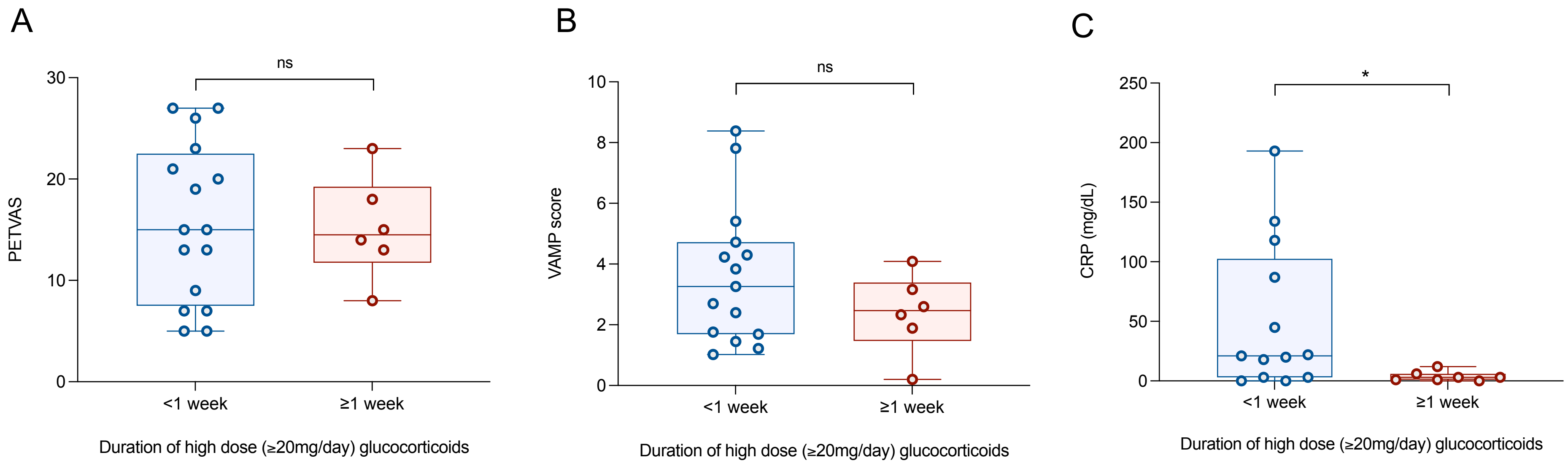

Supplementary figure 8. The effect of high dose glucocorticoids prior to PET/MRI scanning. PETVAS (A), VAMP score (B), and CRP (C) in those treated with high dose glucocorticoids prior to PET/MR scanning *(n*=21). For box and whisker plots, the central line represents the median, the box represents the interquartile range, and the whiskers represent minimum and maximum values. Analysis by two-sided unpaired t-test (PETVAS) and two-sided Mann-Whitney test (CRP). *CRP, C-reactive protein; PETVAS, PET vasculitis activity score.*

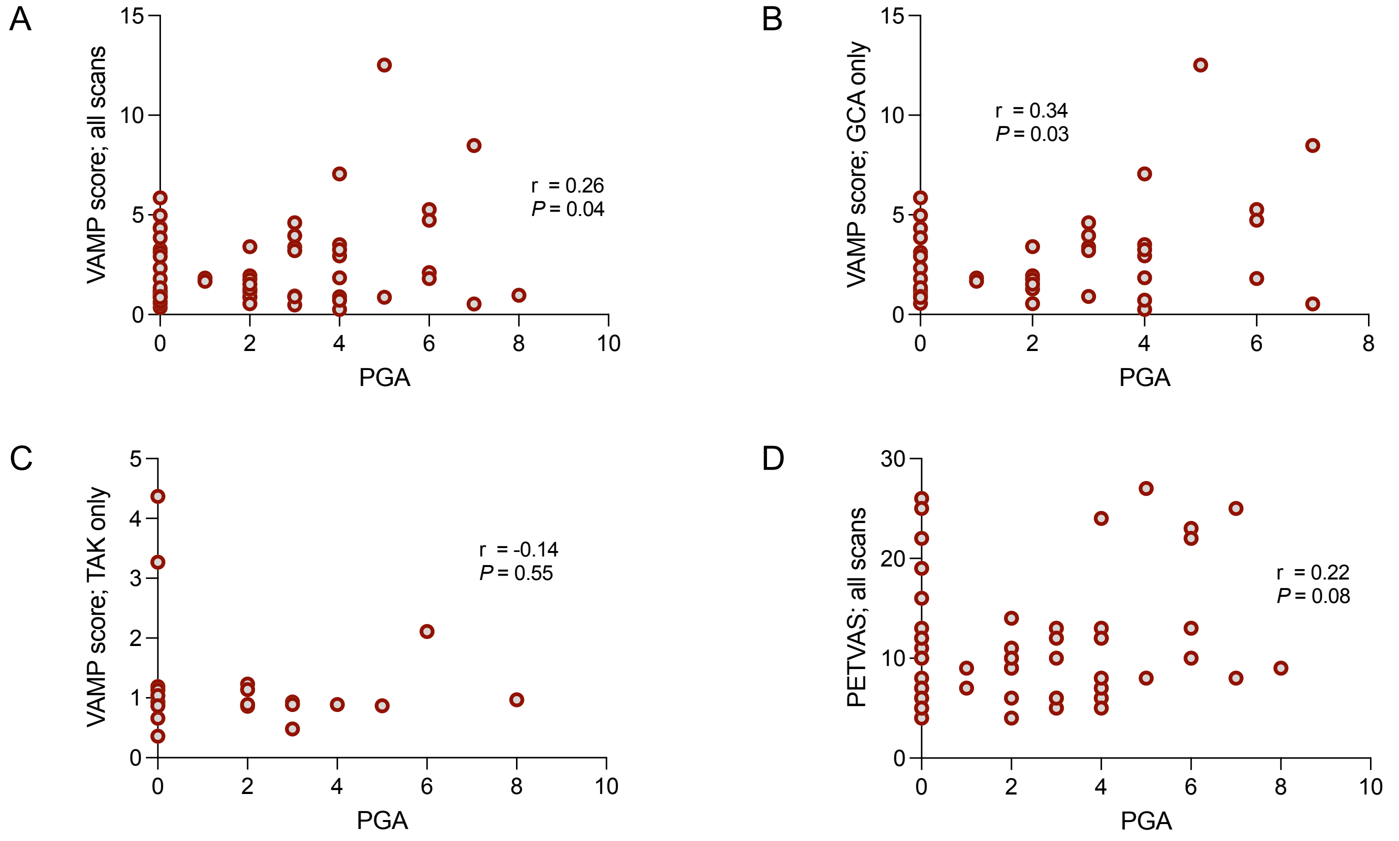

Supplementary figure 9. Validation of the VAMP score. The VAMP score was validated in an independent cohort of 64 LVV PET/MR scans. Correlations are between physician's global assessment (PGA) and VAMP score (A, *P*=0.04), VAMP score (GCA only) (B, *P*=0.03), VAMP score (TAK only) (C), and PETVAS (D). Analysis by two-sided Pearson's correlation coefficient. *GCA, giant cell arteritis; PETVAS, PET vasculitis activity score; PGA, physician's global assessment; TAK, Takayasu arteritis.*

A

B

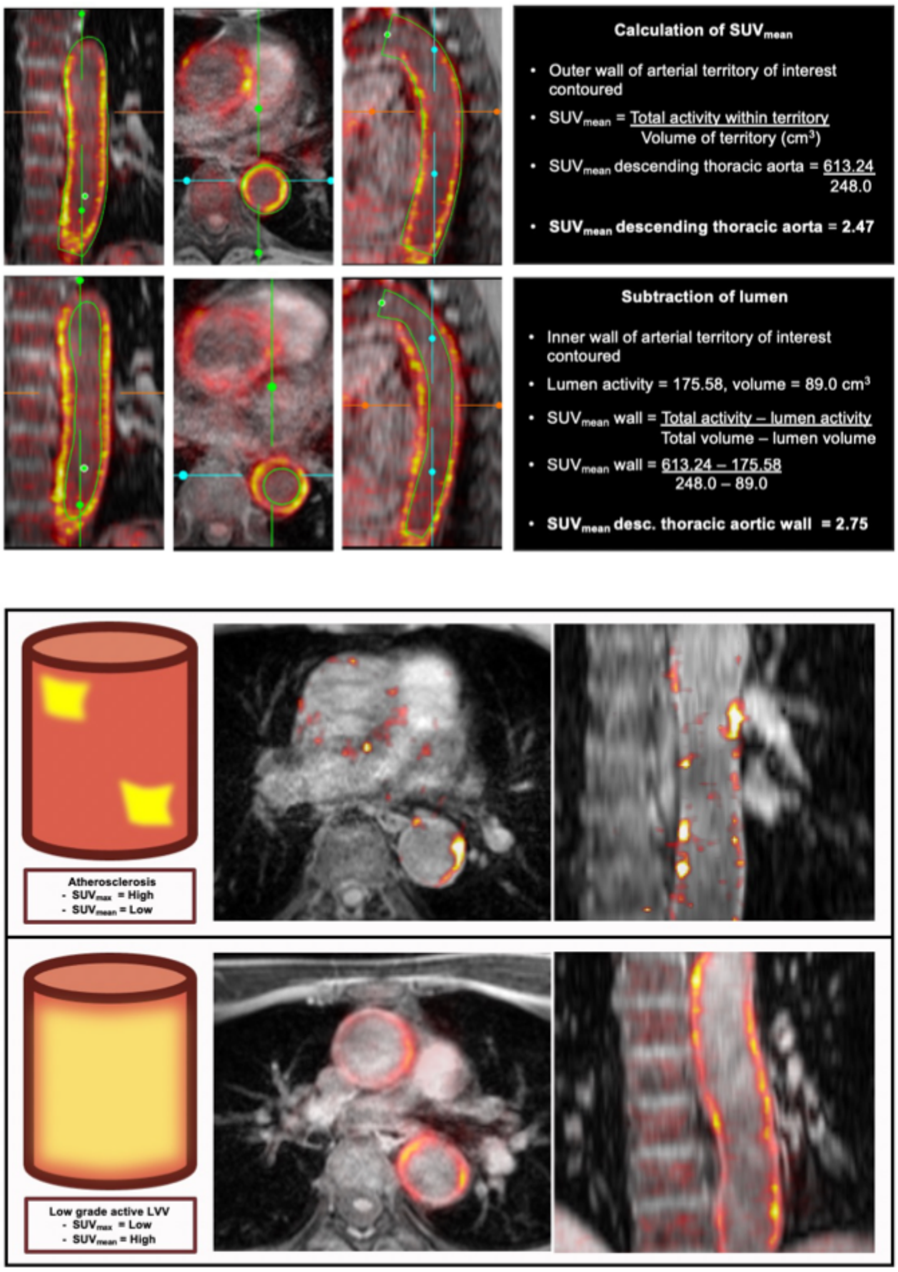

Supplementary figure 10. (A) Example of SUV_{mean} calculation for descending thoracic aorta. (B) SUV_{max} may be prone to misclassification in some instances. The top panel demonstrates a participant with inactive GCA and atherosclerosis. Despite inactive disease, the SUV_{max} was high due to areas of atheroma. SUV_{mean} in the same participant was low and VAMP score correctly indicated inactive disease. The bottom panel demonstrates a participant with low grade active GCA. SUV_{max} in this participant was relatively low despite active disease. SUV_{mean} was, however, high, and VAMP score correctly indicated active disease. *SUV, standardized uptake value.* *SUV, standardized uptake value.*
