# Supplementary material for: 18F-FDG-PET/MR imaging to monitor disease activity in large vessel vasculitis
Source: Nat Commun. 2024 Aug 25;15:7314. doi: 10.1038/s41467-024-51613-1 (PMC11345444; doi:10.1038/s41467-024-51613-1)
Supplement: Supplementary file 3 — Reporting Summary [file 41467_2024_51613_MOESM3_ESM.pdf]

nature portfolio

Corresponding author(s): Prof Neeraj Dhaun

Last updated by author(s): Aug 1, 2024

Reporting Summary

Nature Portfolio wishes to improve the reproducibility of the work that we publish. This form provides structure for consistency and transparency in reporting. For further information on Nature Portfolio policies, see our Editorial Policies and the Editorial Policy Checklist.

Statistics

For all statistical analyses, confirm that the following items are present in the figure legend, table legend, main text, or Methods section.

n/a	Confirmed	
☐	☒	The exact sample size (*n*) for each experimental group/condition, given as a discrete number and unit of measurement
☒	☐	A statement on whether measurements were taken from distinct samples or whether the same sample was measured repeatedly
☐	☒	The statistical test(s) used AND whether they are one- or two-sided<br>*Only common tests should be described solely by name; describe more complex techniques in the Methods section.*
☒	☐	A description of all covariates tested
☐	☒	A description of any assumptions or corrections, such as tests of normality and adjustment for multiple comparisons
☐	☒	A full description of the statistical parameters including central tendency (e.g. means) or other basic estimates (e.g. regression coefficient) AND variation (e.g. standard deviation) or associated estimates of uncertainty (e.g. confidence intervals)
☒	☐	For null hypothesis testing, the test statistic (e.g. *F*, *t*, *r*) with confidence intervals, effect sizes, degrees of freedom and *P* value noted<br>*Give P values as exact values whenever suitable.*
☒	☐	For Bayesian analysis, information on the choice of priors and Markov chain Monte Carlo settings
☒	☐	For hierarchical and complex designs, identification of the appropriate level for tests and full reporting of outcomes
☐	☒	Estimates of effect sizes (e.g. Cohen's *d*, Pearson's *r*), indicating how they were calculated

Our web collection on statistics for biologists contains articles on many of the points above.

Software and code

Policy information about availability of computer code

Data collection	No code used
Data analysis	Imaging data were analyzed using Syngo.via (Siemens, Germany) and FusionQuant (Cedars-Sinai Medical Centre, USA) software. Statistical analyses were performed using Prism (GraphPad Software Inc, USA).

For manuscripts utilizing custom algorithms or software that are central to the research but not yet described in published literature, software must be made available to editors and reviewers. We strongly encourage code deposition in a community repository (e.g. GitHub). See the Nature Portfolio guidelines for submitting code & software for further information.

Data

Policy information about availability of data

All manuscripts must include a data availability statement. This statement should provide the following information, where applicable:

- Accession codes, unique identifiers, or web links for publicly available datasets
- A description of any restrictions on data availability
- For clinical datasets or third party data, please ensure that the statement adheres to our policy

Source data are provided with this paper. De-identified individual participant data are available from corresponding author (bean.dhaun@ed.ac.uk) for up to 6 months from online publication date.

Research involving human participants, their data, or biological material

Policy information about studies with human participants or human data. See also policy information about sex, gender (identity/presentation), and sexual orientation and race, ethnicity and racism.

Reporting on sex and gender	Information provided where available.
Reporting on race, ethnicity, or other socially relevant groupings	Information provided where available.
Population characteristics	Information provided.
Recruitment	Information provided.
Ethics oversight	The study was conducted according to the principles of the Declaration of Helsinki and was approved by the South East Scotland Research Ethics Committee.

Note that full information on the approval of the study protocol must also be provided in the manuscript.

Field-specific reporting

Please select the one below that is the best fit for your research. If you are not sure, read the appropriate sections before making your selection.

[x] Life sciences [ ] Behavioural & social sciences [ ] Ecological, evolutionary & environmental sciences

For a reference copy of the document with all sections, see nature.com/documents/nr-reporting-summary-flat.pdf

Life sciences study design

All studies must disclose on these points even when the disclosure is negative.

Sample size	No specific calculation of sample size was performed for this study. Large vessel vasculitis Is a rare disease, and prospective clinical studies will always be limited by sample size. Indeed, in terms of sample size, our study compares favourably with others in the field. Despite its modest size, our study was able to answer its original question and sets the scene for (ideally) larger and longer studies in the field.
Data exclusions	No data exclusions
Replication	Findings were replicated where possible in an independent cohort of PET/MRI scans from the National institutes for Health, USA. This is outlined within the manuscript.
Randomization	No randomization performed.
Blinding	Reading of PET/MRI scans was blinded, as outlined within the manuscript.

Reporting for specific materials, systems and methods

We require information from authors about some types of materials, experimental systems and methods used in many studies. Here, indicate whether each material, system or method listed is relevant to your study. If you are not sure if a list item applies to your research, read the appropriate section before selecting a response.

Materials & experimental systems

n/a	Involved in the study	
x		Antibodies
x		Eukaryotic cell lines
x		Palaeontology and archaeology
x		Animals and other organisms
	x	Clinical data
x		Dual use research of concern
x		Plants

Methods

n/a	Involved in the study	
x		ChIP-seq
x		Flow cytometry
x		MRI-based neuroimaging

Clinical data

Policy information about clinical studies

All manuscripts should comply with the ICMJE guidelines for publication of clinical research and a completed CONSORT checklist must be included with all submissions.

Clinical trial registration	NCT03914248
Study protocol	Study protocol can be accessed via clinicaltrials.gov with registration NCT03914248
Data collection	Data were collected at the University of Edinburgh between 1st July 2019 and 26th January 2022.
Outcomes	The primary outcome measures were changes in PET/MRI metrics between baseline and follow-up scan (including visual comparison of arterial FDG uptake with liver FDG uptake, SUVmax, SUVmean, mural thickness, mural enhancement, mural signal, and luminal abnormalities).

Plants

Seed stocks	N/A
Novel plant genotypes	N/A
Authentication	N/A
